# Case report: Multiple gastrointestinal perforations in a rare musculocontractural Ehlers–Danlos syndrome with multiple organ dysfunction

**DOI:** 10.3389/fgene.2022.846529

**Published:** 2022-08-15

**Authors:** Huitao Qian, Tao Zhou, Nan Zheng, Qiulun Lu, Yi Han

**Affiliations:** ^1^ The First Affiliated Hospital of Nanjing Medical University, Nanjing Medical University, Nanjing, China; ^2^ Key Laboratory of Cardiovascular and Cerebrovascular Medicine, Collaborative Innovation Center for Cardiovascular Disease Translational Medicine, Nanjing Medical University, Nanjing, China

**Keywords:** Ehlers–Danlos syndrome, CHST14, congenital equinovarus deformity, fragile soft tissues, case report

## Abstract

A 36-year-old male with congenital equinovarus deformity was admitted to the hospital due to worsen deformity. He was known to have ear perforation in childhood. After hospitalization, he received equinovarus correction surgery, fourth toe osteotomy, and external fixation for right foot during the procedure. During his hospital stay, the patient has been treated with multiple gastrointestinal perorations, accompanied with multiple organ dysfunction and fragile soft tissues. During his in-hospital stay, multiple organ dysfunctions were observed, including the heart, kidney, liver, and intestines. In order to identify the mutation site, whole-exome sequencing (WES) was performed, and further verified with Sanger sequencing analysis in this patient. One-site mutation located at *CHST14* [c.883_884del, p (Phe295Cysfs*5)] was identified in this patient, whereas this mutation was not observed in other 100 healthy controls. Also, this variant has not been reported in public databases (ExAC and gnomAD). Our report showed that unanticipated multiple tissue deformation observed the musculocontractural EDS patient was caused by mutation located at *CHST14* [c.883_884del, p (Phe295Cysfs*5)] induced truncated CHST14 protein.

## Introduction

Ehlers–Danlos syndrome (EDS) is a rare hereditary disease, caused by the abnormal development of collagen fibers in connective tissues. It was first reported by a Danish dermatologist Dr. Ehlers, and a French doctor Henri-Alexandre Danlos in 1901. Ehlers–Danlos syndrome is a group of inherited connective tissue diseases demonstrating autosomal dominant, autosomal recessive, and X-linked inheritance patterns (Esaka et al., 2009), with an incidence rate about 1/5,000 (Miller and Grosel, 2020). According to the clinical manifestations, EDSs are mainly categorized into 13 subtypes, including classical type, vascular type, hypermobile type, musculocontractural type, and cardiac-valvular type (Malfait et al., 2017). Despite the different types of EDSs, the common manifestations for EDSs are joint hypermobility, easily bruised soft and elastic skin, and cardiovascular complications in clinics (Beighton et al., 1969; Holzberg et al., 1988; Solomon et al., 1996; Voermans et al., 2009; Malfait, 2018; Mahmood, 2019). Although EDSs were severe and resulted in a short lifespan, the mutations of EDS are preserved and passed down from generation to generation in either autosomal dominant or recessive manner.

Here, we described a rare case of musculocontractural EDSs caused by a single-site genetic mutation in *CHST14* [c.883_884del, p (Phe295Cysfs*5)] accompanied with multiple gastrointestinal perforations and multiple organ dysfunction.

### Patient information

A 36-year-old male with congenital equinovarus deformity was admitted to the hospital because of aggregative pain for more than 1 year. Due to the deformity, he suffered from plaster external fixation surgery for both feet in his childhood. After the surgery, the left foot functioned normally, while the treatment for right foot was terminated due to serious infection. One year later, the pain on the right toe increased after exercise, accompanied with callus formation. When he was 14 years old, he inadvertently fell from high height and suffered from intracranial hematoma oppressing the optic nerve. This eventually developed to nerve atrophy and binocular blindness.

The patient’s right ear perforation occurred at the age of 25, and then debridement and repair were performed. In the next 10 years, several surgeries were performed to treat the recurrent renal calculi, and a stent was implanted for the treatment of urinary tract obstruction.

## Timeline

### Initial admission (October 20 to October 23, 2020, D1 ∼ 4)

**FIGURE 1:**
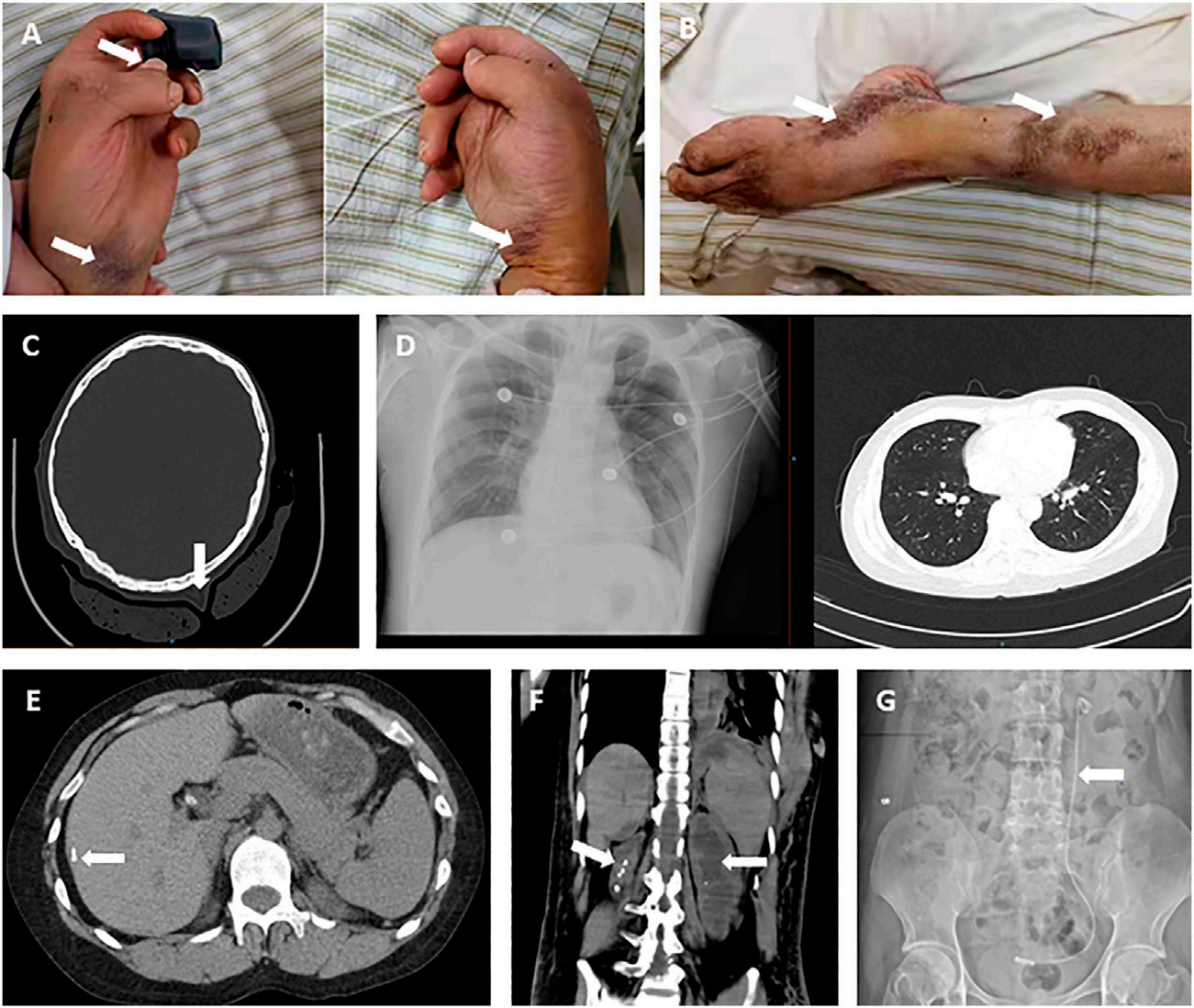
Patient’s clinical images at admission. **(A)** Claw-shaped hands with multi-finger deformity of the left thumb, with easily bruised and over stretchable skin (arrow). **(B)** Foot drop deformity (left), with surgical scars on the skin of the leg. **(C)** Over stretchable soft tissue of head shown on CT image. **(D)** X-ray images were presented for the lung in normal shape and size. **(E)** Calcifications in the liver. **(F)** Kidney atrophy (right), hydronephrosis, and renal calculi. **(G)** J stent in the urinary tract (left).

At admission, physical examination was performed, identifying right foot (about 110°), calcaneal internal rotation (about 20°), forefoot adduction (about 30°), varus (about 15°), shortening of right toe, and mild drooping of left foot with mild varus, indicating the typical manifestations of metatarsal flexion. The markers from previous surgeries were easily observed, including surgical scar, pigmentation, muscle atrophy, and valgus deformity of both feet, accompanied with callus formation on the sole of the right forefoot. In addition, metacarpal curvature of hands (about 20°) and multi-finger deformity of the left thumb were observed (Figure 1). The skin was soft and over stretched.

In detail, the active and passive movements of the right toe were limited (about -10°–5°). The passive traction pain was identified on the right ankle joint and the fourth toe of the right foot. Both adduction and varus occurred on the right foot with muscle strength of grade 3. The active movement of the left ankle was also limited (about -10°–15°), with equinovarus deformation and muscle strength of grade 4. In addition, the dorsal extension was observed between both hands. Both lower limbs and upper limbs were normal.

The results from digital radiography at the right ankle confirmed the deformity as equinovarus, reflecting from the interosseous joints being angled, and part of the joint space narrowed, with possible osteoarthritis. On October 19, 2020, computed tomography (CT) scan was performed and found multiple solid nodules in both lungs, calcification in the right lobe of the liver, cholecystolithiasis, splenomegaly, right kidney atrophy, bilateral nephrohydrosis and renal calculi, implantation of left ureteral stent, and focal calcification of the prostate (Figure 1).

The primary diagnosis for this patient was 1) equinovarus (bilateral); 2) claw-shaped hands (bilateral); 3) blind (binocular); 4) cholecystolithiasis; 5) nephrohydrosis (bilateral); and 6) renal calculi (bilateral). Clinical laboratory findings over the treatment course of this patient for deformity correction and gastrointestinal perforations are shown in Table 1 phase I.

**TABLE 1:** | Clinical laboratory findings over the treatment course of EDS and gastrointestinal perforations.

	2	9	15	16	17	18	19	20	21	22	23	25	26	27	28	30	33	35	36	37	38
Day of illness																					
WBC (10^9/L)	5.42	7.75	2.89	5.63	14.13	18.32	11.67	8.82	5.82	9.42	14.67	16.83	54.48	26.88	18.91	14.59	11.86	10.59	15.03	13.6	—
Ne (% )	54.45	75.47	81.4	82.7	94.1	94.6	92.2	88	76.2	79.9	75.5	86.53	86.8	84	87.3	87.2	84.13	76.2	83.2	84.4	—
Hb (g/L)	127	164	123	119	93	98	93	99	89	103	78	68	117	68	72	71	74	99	101	81	—
Plt (10^9/L)	252	323	251	326	129	104	78	81	88	162	219	289	567	275	209	194	233	194	183	124	—
Urea (mmol/L)	3.77	—	8.78	8.66	6.93	6.95	9.99	—	14.3	—	—	7.6	14.11	13.43	12.6	10.04	14.25	24.13	23.52	29.9	—
Cre (umol/L)	72.3	—	120.7	138.8	122.6	107.4	81.5	—	98.5	—	—	93.1	239.7	199.4	191.2	131.1	117.8	118.1	117.9	181.6	—
AST (U/L)	19.5	—	101.8	45	31.6	—	19.5	—	15.2	—	—	33.5	17	22.4	24.5	25.6	28.6	30.1	64.2	51.8	—
ALT(U/L)	16.4	—	102.3	34.2	30.1	—	24.3	—	8.6	—	—	24	36.8	19.5	6.2	16.3	19.1	11	31.2	37	—
CK-MB (ng/mL09;	—	—	—	2.41	—	2.59	—	—	—	—	0.644	—	1.56	1.28	1.91	0.178	0.594	1.12	1.59	3.61	3.06
cTNT (ng/L)	—	—	—	53.14	—	30.55	—	—	—	—	63.69	—	100.5	72.71	52.93	39.1	36.72	38.88	37.1	38.47	38.36
MYO (ng/mL)	—	—	—	1062	—	361.5	—	—	—	—	164.6	—	753.5	747.1	514.4	224.9	148.6	299	373.6	418.7	285.6
ProBNP (pg/mL)	—	—	—	10129	15525	9932	2735	—	—	413.4	1068	—	3521	1920	1119	971.4	552.3	138.4	400.1	1109	509.9
PCT (ng/mL)	—	—	—	>100	>100	>100	76.38	41.53	21.73	—	—	—	22.35	14.71	7.77	2.88	1.19	8.07	4.37	4.23	—
CRP (mg/L)	—	—	—	—	—	—	—	—	—	—	—	—	—	—	260	—	—	84.6	—	—	—

### Equinovarus orthopedics (October 23 to November 2, 2020, D5 ∼ 15).

**FIGURE 2:**
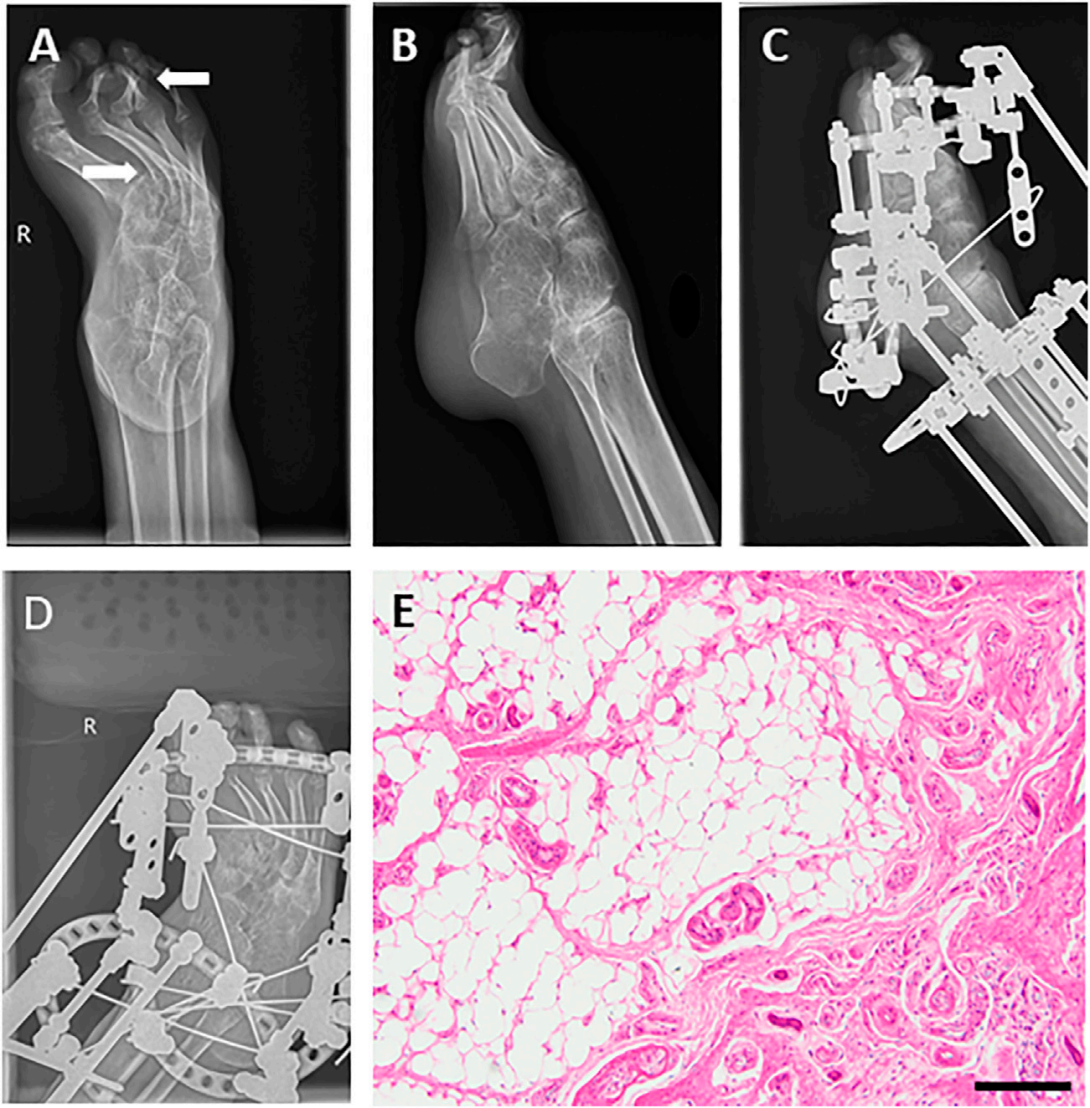
Images before and after orthopedics for equinovarus deformity of the right foot. **(A,B)** Foot drop and equinovarus deformity on DR (right). **(C,D)** Images after orthopedics of equinovarus deformity (right). **(E)** Pathological section of the fourth toe of the right foot showed chronic mucosal inflammation with submucosal fibrous adipose tissue hyperplasia and a little cortical bone tissue. Bar = 200 μm.

The equinovarus correction surgery was performed including fourth toe osteotomy and external fixation for the right foot. In this surgery, thin skin, obvious degeneration of subcutaneous fat and muscle were further confirmed, and the right Achilles tendon was extended. Regardless of infection from the incision, the right ankle still took a long recovery time (Figure 2). The results of postoperative laboratory detection are shown in Table 1 phase II.

### Colonic perforation (November 3 to November 12, 2020, D16 ∼ 25)

**FIGURE 3:**
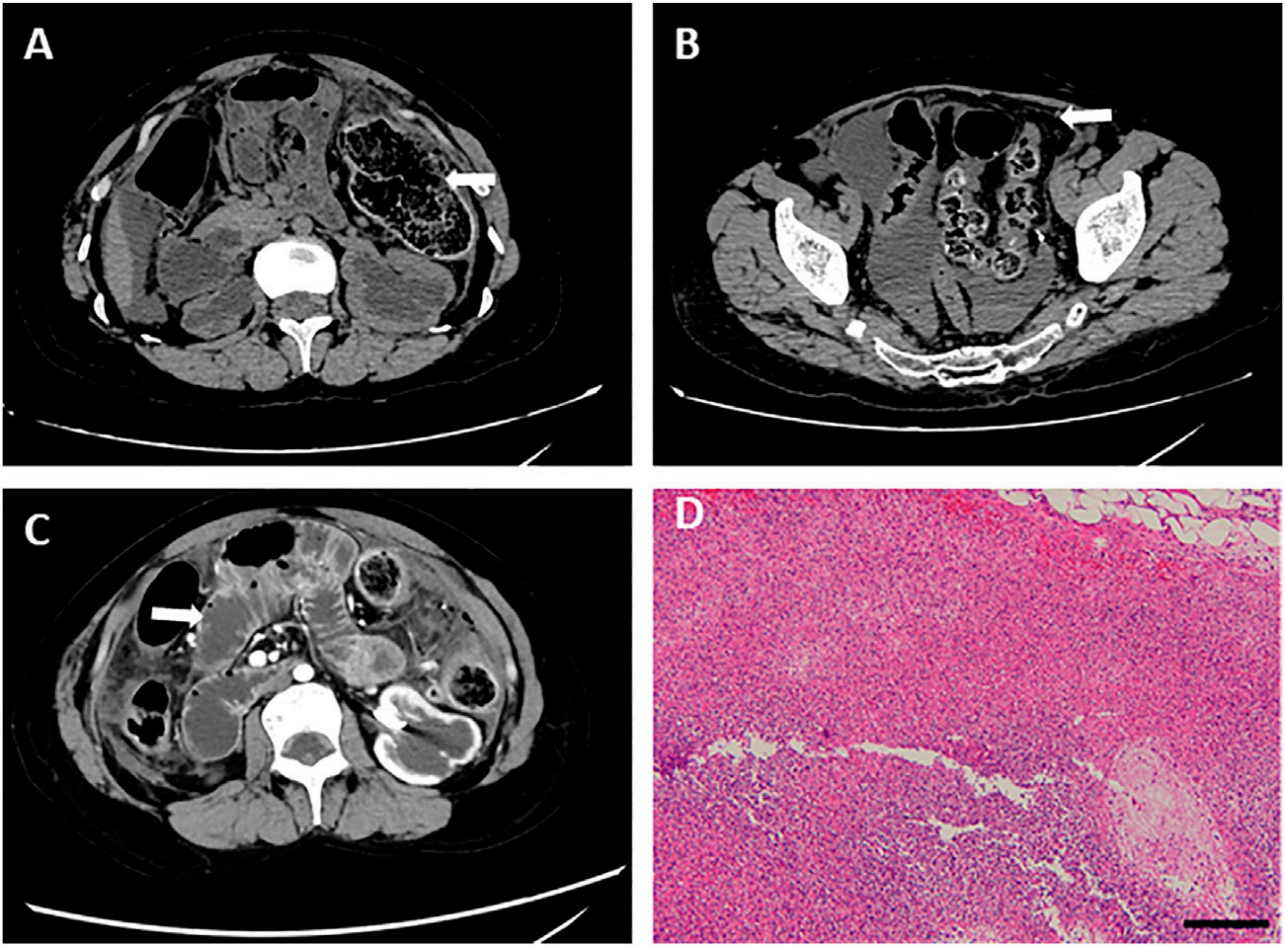
Abdominal CT images before the operation for intestinal perforation and pathological detection. **(A)** Intestinal wall of the splenic flexure of the colon was discontinuous. **(B)** Abdominal and pelvic effusion. **(C)** Dilated duodenum and jejunum. **(D)** HE staining was performed using splenic flexure of the colon, showing a large number of inflammatory cell infiltration and abscess formation. Bar = 200 μm.

The patient felt severe abdominal pain on 3 November 2020. Due to the observation of abdominal distention and tenderness, an abdominal CT scan was performed, revealing intestinal perforation, intestinal obstruction, and peritonitis. In detail, the intestinal wall of the splenic flexure of the colon was discontinuous and surrounded by abdominal and pelvic effusion mixed with intestinal contents (Figure 3). The duodenum and jejunum were dilated, accompanied by the thickened colon wall and contents accumulation in the intestines, indicating it is feasible for intestinal obstruction.

Substantially, emergency laparotomy plus partial colectomy was performed. A large number of turbid ascites and fecal residue were observed in the abdominal and pelvic cavities. In the splenic curvature of the colon, necrotic perforation was identified. In addition, fecal overflow was observed from the perforation, and blood circulation was insufficient at the proximal colon (around 15 cm), resulting in necrosis of relevant intestines. Partial colectomy and proximal fistulotomy were performed.

After the operation, he was attacked by septic shock with hypotension and high heart rates, and then transferred to ICU for further supportive treatment. At the beginning of his ICU stay, his body temperature increased to 39 °C, and laboratory detection found WBC 18.3 × 10^9, PCT>100 ng/ml, ProBNP 15,525 pg/ml, indicating severe infection and cardiac injury. Therefore, anti-infection therapy, nutritional support, blood transfusion, and mechanical ventilation were used. Once the vital signs were gradually stabilized, the patient was weaned from mechanical ventilation and moved back to the general ward. Clinical images of the patient for this phase are shown in Figure 3. Laboratory findings over this treatment course are displayed in Table 1 phase III.

### Gastric perforation and further intestinal perforation (November 13 to November 26, 2020, D26∼39)

**FIGURE 4:**
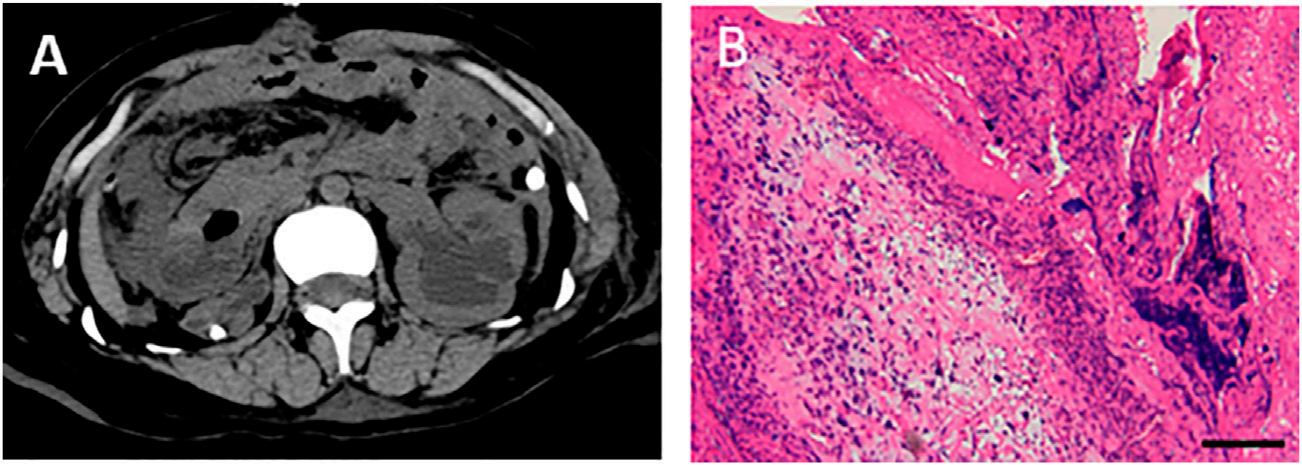
Abdominal CT images after gastric perforation and the pathological detection for gastric tissues. **(A)** Abdominal CT image after gastric perforation. **(B)** HE staining was performed with gastric tissues, showing inflammatory cells infiltrated and fibroblasts proliferated in the fibrous connective tissue of gastric tissue at pyloric perforation. Bar = 200 μm.

On day 26, abdominal drainage for this patient was dark brown, indicating one more shoot of gastrointestinal perforation. In this way, an emergency exploratory laparotomy was performed, finding a 5-cm perforation in the anterior wall of the gastric antrum, with valgus of the crisp and edematous gastric mucosa, turbid ascites, and extensive adhesion of the small intestine, edematic, and brittle intestines. The pathological section images of gastric tissue are presented in Figure 4. After gastric perforation, the abdominal CT images were performed (Figure 4), identifying inflammatory peritoneal effusion and exudation. In addition, the patient was attacked by another round of septic shock, and then took a slow recovery with antibiotic and organ supportive treatment. One more week later, the abdominal drainage was from clear light yellow to turbid dark green, indicating another possible intestinal perforation on November 21. Because of multiple organ dysfunction, further surgery was not considered. Clinical images of the patient for this phase are shown in Figure 4. Laboratory findings over this treatment course are displayed in Table 1 phase IV.

On November 26, the patient was infected by third round of shock due to sepsis, resulting in multiple organ dysfunction. Since then, his vital signs and organ functions collapsed drastically, and finally dead.

### Identification of genomic mutation in this patient

**FIGURE 5:**
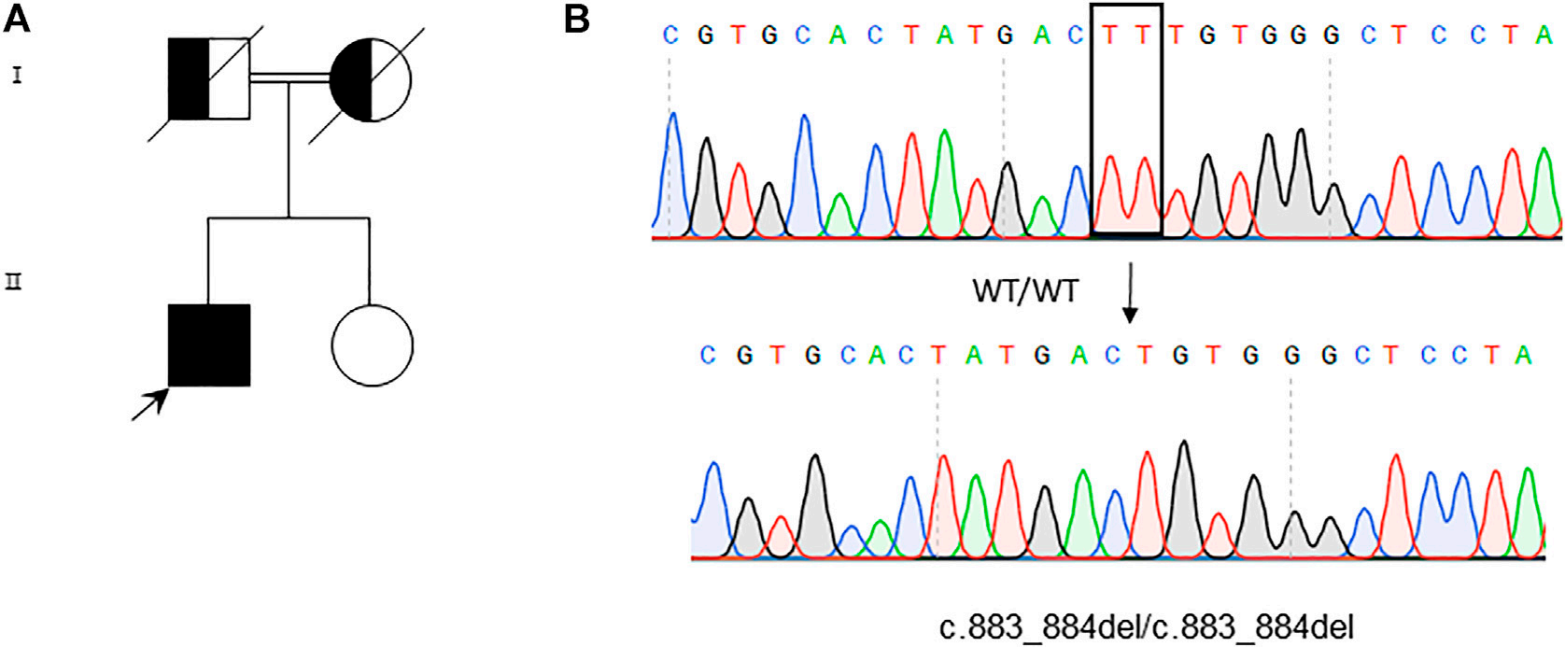
Whole-exome sequencing and variants verification by Sanger sequencing. **(A)** Genogram of the patient. **(B)** Sequence analysis reveals homozygous TT deletion at 883 and 884 of the *CHST14* gene in the patient. The deleted nucleotides are boxed in the WT sequence (upper panel), while the arrow indicates the position of the deleted nucleotides in the mutation sequence (lower panel).

To explore whether this patient suffered with musculocontractural Ehlers–Danlos syndrome is caused by genetic defect or not, the genome for this patient was analyzed with whole-exome sequencing (WES) analysis. We found a homozygous deletion of T at position 883 and 884 of *CHST14*, resulting in the substitution of phenylalanine at position 295 by cysteine of *CHST14* [c.883_884del, p (Phe295Cysfs*5)] (Figure 5). This result was further verified by Sanger sequencing (Figure 5). Furthermore, we were not able to detect the same mutation of *CHST14* among 100 healthy controls. According to the diagnostic criteria of mcEDS, we further analyzed the consistency of this case, as shown in Table 2.

**TABLE 2:** Diagnostic criteria of mcEDS.

Major criteria	Minor criteria
Adduction–flexion contractures	Recurrent/chronic dislocations
Talipes equinovarus	Pectus deformities
Distinct facial features	Scoliosis/kyphoscoliosis
Skin hyperextensibility	Digital tapering, slender, and cylindrical
Skin fragility	Progressive talipes deformities
Increased palmar wrinkling	Large subcutaneous hematomas
—	Chronic constipation
—	Colonic diverticula
—	Pneumothorax/pneumohemothorax
—	Nephrolithiasis/cystolithiasis
—	Hydronephrosis
—	Cryptorchidism in males
—	Strabismus
—	Myopia/astigmatism
—	Glaucoma

## Methods

### Whole-exome sequencing

Exome sequencing: exome sequencing was performed using a full exome capture chip, which was sequenced by Illumina series sequencer. The target sequence coverage was not less than 99%.

Gene data analysis: systematic analysis and screening of the cloud platform for accurate diagnosis of genetic diseases were applied for this case. We used the Genome Analysis Toolkit (GATK) and Unified Genotyper algorithm with the dataset of dbsnp137 (java -jar GenomeAnalysisTK.jar -T Unified Genotyper). Substantially, the detected genetic variants were annotated by ANNOVAR using gene models, population frequencies, and mutation severity prediction.

### Verification of suspected pathogenic mutation

The mutation site identified using WES was verified by Sanger sequencing in the patient. The blood from cohorts was centrifuged, and DNA was extracted by TIANamp Genomic DNA Kit. Sequences for the PCR reaction were as following: forward: 5′- GAT​GTC​ACA​TTC​CCC​GAG​TT-3’; reverse: 5′- AGT​GGT​AAT​GCA​GGC​TTT​CG -3’. The site mutation was also sequenced with 100 healthy controls.

## Discussion

EDS, a congenital disorder, is mainly presented in the abnormal connective tissue system including the structure, production, and processing of collagens (Malfait et al., 2020). In this case, we find an example of gastrointestinal perforations in mcEDS and a novel genetic mutation site of CHST14 [c.883_884del, p (Phe295Cysfs*5)], which can cause musculocontractural EDS.

In this case, the patient is presented with congenital equinovarus, accompanied by obvious abnormalities of craniofacial bone, skin, and musculoskeletal bone and was hospitalized several times because of kidney stones and renal atrophy. In previous studies, there were only two genes *CHST14* and *DSE,* reported to be associated with mcEDS (Miyake et al., 2010; Müller et al., 2013; Schirwani et al., 2020). After WES analysis, a homozygous mutation of *CHST14* [c.883_884del, p (Phe295Cysfs*5)] was identified in this patient, which has not been reported. *CHST14* is a single exon gene that encodes glycosylsulfonyltransferase 14, which can catalyze the transformation of chondroitin sulfate (CS) to dermatan sulfate (DS), one kind of glycosaminoglycan (GAG). Mostly, DS is covalently linked to dermatan sulfate proteoglycan (DSPG) *via* its’ terminal xylose and serine residues. DSPG is universally expressed in vascular wall, cornea, skin, tendons, cartilage, bone, and undifferentiated mesenchymal tissues. It participated in different pathophysiological processes such as cardiovascular disease, tumorigenesis, inflammation, injury repair, and fibrosis (Malfait et al., 2010; Janecke et al., 2016; Mizumoto et al., 2017). *CHST14* mutation decreases DSPG levels, affecting the collagen formation and fibers structure. Up to now, 66 patients from 48 families with mcEDS- *CHST14* and five patients with mcEDS-*DSE* from four families have been identified (Kosho et al., 2019; Minatogawa et al., 2021).

In this case, the patient died of infection caused by gastrointestinal perforation eventually. Generally, intestinal obstruction and inflammatory bowel disease are common causes of non-traumatic intestinal perforation. It is possible that the patient’s long-term bed rest after orthopedic surgery causes the weakening of gastrointestinal peristalsis and secondary intestinal obstruction, which in turn worsens intestinal perforation. Combined with his special manifestations, we suspected that the perforation is caused by tissue and muscle fragility.

The limitation for this study is the missing genetic information from his family. However, there is no positive or typical manifestation for his parents.

The diagnosis of EDS is mainly established on clinical manifestations, pedigree analysis, biochemical examination, and genetic analysis. Clinicians can usually infer the diagnosis of EDS, according to the patient’s clinical manifestation and medical history, whereas the definite diagnosis still requires genetic examination. Therefore, it is essential for clinicians to do genetic screening for suspected patients.

## Data Availability

The original contributions presented in the study are included in the article/ Supplementary Material, further inquiries can be directed to the corresponding author/s.

## Abbreviations

WBC, white blood cell; Ne%, Neutrophil percentage; Hb, Hemoglobin; Plt, Platelet; Cre, Creatinine; AST, Aspartate aminotransferase; ALT, Alanine aminotransferase; CK-MB, Creatine Kinase, MB Form; cTNT, cardiac Troponin T; MYO, Myoglobin; ProBNP, Pro Brain Natriuretic Peptide; PCT, Procalcitonin; CRP; C-reactive protein.
